# Platelet activating factor levels and metabolism in tangier disease: a case study

**DOI:** 10.1186/1476-511X-11-89

**Published:** 2012-07-08

**Authors:** Vana Kolovou, Vasiliki D Papakonstantinou, George Stamatakis, Sophia N Verouti, Marianna N Xanthopoulou, Genovefa Kolovou, Constantinos A Demopoulos

**Affiliations:** 1 Cardiology Department and Molecular Immunology Laboratory, Onassis Cardiac Surgery Center, Athens, Greece; 2 Biochemistry Laboratory, Faculty of Chemistry, National and Kapodistrian University of Athens, Athens, Greece; 3 Department of Science of Nutrition-Dietetics, Harokopio University, Athens, Greece

**Keywords:** PAF, Tangier Disease, Atherosclerosis, Lp-PLA_2_, PAF-AH, Lyso-PAF-AT, PAF-CPT

## Abstract

**Background:**

Tangier disease (TD) is a phenotypic expression of rare familial syndrome with mutations in the ABCA1 transporter. The risk of coronary artery disease in patients with TD is variable. On the other hand the pivotal role of Platelet-Activating Factor (PAF) mediator in atheromatosis was found. Plasma lipoproteins are transporters of the PAF acetylhydrolase (PAF-AH) in cells and known as lipoprotein-phospholipase A_2_ (Lp-PLA_2_) in plasma and regulators of PAF levels in blood. In addition, PAF can be biosynthesized from the remodeling and the *de novo* pathways in which Lyso-platelet activating factor-acetyltransferase (Lyso-PAF-AT) and platelet activating factor-cholinephosphotransferase (PAF-CPT) are the regulatory enzymes. The aim of this study is to investigate in a TD patient with a unique mutation (C2033A), the concentration of PAF in blood, the Equivalent Concentration for 50% aggregation (EC_50_) values of platelet rich plasma (PRP) toward PAF, adenosine diphosphate (ADP) and thrombin, and the activities of PAF metabolic enzymes Lp-PLA_2_, PAF-AH, Lyso-PAF-AT and PAF-CPT.

**Methods:**

The EC_50_ value of PRP was measured by an aggregometer. The determination of the specific activity of PAF-CPT and Lyso-PAF-AT was made after *in vitro* enzymatic assay, chromatographic separation and measurement of the produced PAF in a biological assay with washed rabbit platelets. The determination of PAF-AH and Lp-PLA_2_ was made after an *in vitro* enzymatic assay from the decay of radioactive PAF.

**Results:**

The TD patient had lower bound-PAF values in blood, decreased specific activity of PAF-CPT and Lyso-PAF-AT, increased specific activity of PAF-AH in platelets and leukocytes and Lp-PLA_2_ activity in plasma compared to healthy women. The EC_50_ of PAF and Thrombin were higher compared to healthy women.

**Conclusion:**

The increased Lp-PLA_2_ activity, as well as, the decreased activities of PAF-CPT and Lyso-PAF-AT, explain the decreased bound-PAF level in TD patient and the EC_50_ of PAF. However, total PAF is in a normal range and this probably can explain one of the reasons this TD patient has no CAD.

## Introduction

Tangier disease (TD) is a rare genetic disorder that was first described by Fredrickson et al. [1] and it is characterized by nearly absence of high density lipoprotein (HDL) cholesterol in patients’ plasma. Only recently, in 1999, has been reported that mutations in the ATP binding cassette A1 transporter (ABCA1) is responsible for TD [2-4].

ABCA1 is a member of the ABCA subfamily with high expression levels in hepatocytes, adrenal glands, liver, lung and several other tissues [5,6]. *In vivo* models with ABCA1 inactivation demonstrated cholesterol deposition in macrophages and other cells [7], suggesting a pivotal role of this transporter in the trafficking of lipids, HDL biogenesis, and overall cholesterol homeostasis.

Approximately, 1/3 of TD patients may develop coronary artery disease (CAD) [8]. The mechanism of CAD in TD patients is still not very clear. Several mechanisms, beside lipid metabolism, may be involved in the development of CAD. Inflammation is the one of these mechanisms. The inflammation is initiated by leukocytes and lipoproteins infiltration into the arterial wall (1), where the lipoproteins undergo oxidation (2) and the monocyte-derived macrophages after digestion of modified lipoprotein particles form foam cells [9]. Macrophages, after their activation, synthesize proinflammatory and prothrombotic factors such as platelet-activating factor (PAF) [10] which is a powerful mediator of inflammation [11] and a key factor for atherosclerosis [12]. A pathological regulation of PAF metabolism results in increased PAF-levels and triggers local inflammatory response of vascular endothelium [12].

PAF biosynthesis is regulated by two different enzymatic pathways: the *de novo* pathway in which the key enzyme is specific dithiothreitol-insensitive CDP-choline: 1-alkyl-2-acetyl-sn-glycerolcholinephosphotransferase (PAF- cholinephosphotransferase, PAF-CPT, EC 2.7.8.16) that converts 1-O-alkyl-2-acetyl-glycerol to PAF; and the remodeling one in which the key enzyme is Lyso-PAF:acetyl-CoA acetyltransferase (Lyso-PAF-acetyltransferase, Lyso-PAF-AT, EC 2.3.1.67) [13] which acetylates Lyso-PAF. It is believed that the *de novo* reaction sequence appears to be responsible for its constitutive synthesis maintaining resting PAF levels in various tissues and blood whereas the remodeling route plays a crucial role in inflammatory/hypersensitivity responses of PAF.

PAF is catabolized by PAF-acetylhydrolase (PAF-AH, EC 3.1.1.47). PAF-AH has an isoform in plasma, also known as lipoprotein-associated phospholipase A2 (Lp-PLA_2_) [14]. PAF-AH action is to cleave short chain acyl chains at the *sn-*2 position of phospholipids [14] such as oxidized phospholipids and PAF [10]. Since PAF-AH cleaves PAF (which is an atherogenic factor) it can be characterized as an anti-atherogenic enzyme [15].

In the present study, we determined in a TD patient the specific activities of PAF anabolic enzymes (PAF-CPT and Lyso-PAF-AT) in platelets and leukocytes as well as the specific activity of the catabolic enzyme PAF-AH in the same set of cells and erythrocytes along with the activity of Lp-PLA_2_ in plasma. We measured PAF levels (free, bound and total) in blood and we studied the EC_50_ values of PAF, thrombin and adenosine diphosphate (ADP) in Platelet-Rich Plasma (PRP).

## Patients and methods

### Patient with TD

We studied a 43 years old Greek women with TD, previously described by Kolovou et al. [16]. The patient had no CAD documented angiographically [17] and had no signs of carotid artery atherosclerosis as evaluated by echocardiography. At the present time she was an ex-smoker. She is the child of two second-degree cousins and a mother of three girls. Clinical examination revealed mild hepatosplenomegaly. Blood samples were performed in the 21st day of her menstrual cycle and the lipid measurements were made in the same day. No enlarged tonsils, pathological lymph nodes, peripheral neuropathy or atherosclerosis were present. Her lipid profile was typical of TD phenotype. DNA analysis has revealed a new mutation (C2033A) in both alleles, causing a premature stop codon at the amino acid residue 573, which resulted in truncation of the ABCA1 protein [18].

### Control group

The control group consisted of 12 women aged 64 ± 10 years old who were self-reported as healthy.

The Department of Chemistry of National and Kapodistrian University of Athens and Onassis Cardiac Surgery Center ethics committee approved the protocol of this study. All subjects signed an informed consent form.

#### Isolation of human PRP and PPP (platelet poor plasma) for the determination of EC50 values

Human blood, collected from an antecubital vein, was distributed into 2 polyethylene tubes containing anticoagulant (0.1 M buffered dextrose citrate, ACD) in the ratio of blood/anticoagulant: 9/1 (v/v) to a final volume of 10 mL. The isolation of PRP was performed as previously described [19,20]. Briefly, the PRP was obtained by centrifugation (1st) of blood specimens at 194 × g for 10 min. PRP was then transferred to polypropylene tubes at room temperature for the biological assay, whereas PPP was obtained by further centrifuging (2nd) the specimens at 1465 × g for 20 min. PRP was adjusted to 500000 platelets/μL using the respective PPP. All procedures took place at 24 °C (room temperature).

#### Isolation of human plasma, platelets, erythrocytes and leukocytes

The isolation was performed as previously described [21]. Briefly, human blood, collected from an antecubital vein was distributed into 1 polyethylene tube containing anticoagulant in the ratio of blood/anticoagulant: 9/1, so as the total volume in each tube to be 10 mL. The procedure is the same as above but after the 2nd centrifugation (1465 × g, 20 min) the supernatant (plasma) was collected, aliquoted and stored at -80°C. In the pellet from the 2nd centrifugation 1 mL of a buffer containing 50 mM Tris–HCl (pH 7.4) was added and was sonicated 4× 15 s in an ice bath, the sample was then centrifuged (3rd) at 500 × g for 10 min at 4°C. The supernatant was the platelets homogenate which was aliquoted and stored at -80°C. In the pellet of the 1st centrifugation saline to a final volume of 10 mL was added. After mildly redissolving, 3.4 mL of dextran solution was added (3% dextran in NaCl 0.15 M) to induce erythrocyte sedimentation. The mixture was kept for 1 hour at room temperature and the leukocyte-rich supernatant (LRP) was then centrifuged (4th) at 500 × g for 10 min at room temperature. The remaining erythrocytes in the sediment were lysed with the addition of 5 mL of a lyses solution consisted of 155 mM NH_4_Cl, 10 mM KHCO_3_, and 0.1 mM EDTA, and after 5 min removed by centrifugation (5th) at 300 × g for 10 min at room temperature. The pelleted cells (leukocytes) were resuspended in 1 mL of a buffer containing 50 mM Tris–HCl (pH 7.4) and then sonicated into ice for 4 × 15 s. After a centrifugation (6th) at 500 × g for 10 min at 4°C the supernantant of washed human leukocytes (WHLs) homogenate was aliquoted and stored at -80°C. From the sediment of erythrocytes a volume of 500 μL was added in 2.5 mL of saline and then centrifuged (7th) at 200 × g for 10 min at room temperature. The pellet was resuspended in 2 mL of a buffer containing 50 mM Tris–HCl (pH 7.4) and centrifuged at 500 × g for 10 min at 4°C. The supernatant was aliquoted and stored at -80°C.

#### Human PRP aggregation studies

Human PRP was obtained as previously described. Aliquots of PAF solution in chloroform/methanol (1:1 v/v) were evaporated under a stream of nitrogen and were redissolved in BSA (1.25% in saline) to obtain PAF solutions with final concentrations into cuvette ranging from 1.0 10^–8^ to 1.0 10^–5^ mol/L. ADP and Thrombin were dissolved in saline to obtain solutions with final concentrations into cuvette ranging from 10^-10^ to 10^-9^ M of ADP and 90 to 370 U/L of Thrombin, respectively. The maximum reversible or the minimum irreversible PAF-induced platelet aggregation was determined as the 100% aggregation, and then various PAF concentrations were added, so as to achieve aggregations between 20% and 80%. These PAF-induced aggregations were of linear response to the respective PAF concentration; therefore, the EC_50_ value was calculated. EC_50_ (Equivalent Concentration for 50% aggregation) accounts for the aggregating agent concentration inducing 50% aggregation [21,22] and results are expressed as M or Units (final concentration in the cuvette).

#### PAF isolation and purification

The isolation and purification of PAF was according to the method of Demopoulos et al. [23]. Briefly, 10 mL of blood were collected from each human subject and poured immediately (after collection) into 40 mL of absolute ethanol. The mixture was stirred and centrifuged at 300 × g for 10 min. The supernatant and the pellet were extracted separately according to the Bligh and Dyer method [24] and the chloroform phase in each case was stored. The supernatant chloroform extract contains plasma PAF, named as free PAF, while the pellet extract contains cell–PAF, named as bound PAF. The above extracts were purified on silicic acid column chromatography that was eluted with 45 mL of methanol/water (1:1.5, v/v), followed by 50 mL of methanol/water (2:1, v/v). The initial 45 mL (containing the bulk of proteinaceous and other non-lipid impurities) were discarded while the PAF containing eluents were further purified by HPLC on a cation-exchange column. The solvent system consisted of an isocratic elution of acetonitrile/methanol/water (61:31:8, v/v/v) slightly modified from the one described in [23]. The eluted substances were detected using UV detection at 208 nm.

#### Washed rabbit platelet aggregation

Washed rabbit platelets were prepared as previously described [10]. PAF and the all examined samples were dissolved in BSA 1.25% in saline. The PAF concentration of the samples was estimated against an 8 point regression curve of standard PAF. The study was performed using a Chronolog aggregometer (model 400) (Havertown, Pa, USA) at 37°C with constant stirring at 1200 rpm, coupled to a Chronolog recorder.

#### PAF-AH activity assay

The PAF-AH enzymatic assay was performed as previously described [25]. Briefly, Tris–HCl 50 mM pH 7.4 buffer was mixed with 4 nmol of ^3^ H]-PAF (20 Bq per nmol)/PAF solution in BSA (1% in saline). The whole mixture was incubated at 37°C for 5 min and the reaction was initiated by adding homogenates (0.25 mg/mL for leukocytes, 0.5 mg/mL for platelets and 2.5 mg/mL for erythrocytes at final volume of 200μL). The reaction took place at 37°C for 30 min and was stopped by the addition of BSA solution (final concentration 0.75 mg/mL) and followed by precipitation with trichloroacetic acid (TCA; final concentration 9.6% v/v). The mixture stayed in ice bath for 30 min and then was centrifuged at 16000 × g for 5 min at 4°C. The ^3^ H]-acetate released from the reaction was measured on the liquid scintillation counter by placing 0.1 mL of the suspension liquid into 5.0 mL of scintillation liquid. The enzyme activity was expressed as pmol of PAF degraded per minute per mg total protein except of the fraction of plasma where the enzyme activity was expressed as pmol of PAF degraded per min per μL of plasma. All experiments were conducted in triplicates.

#### Dithiothreitol-insensitive PAF-CPT activity assay

Assay was performed at 37°C for 20 min in a final volume of 200 mL containing 0.05 mg/mL protein, 100 mM Tris–HCl (pH 8.0), 15 mM DTT, 0.5 mM ethylenediaminetetraacetic acid (EDTA), 20 mM MgCl_2_, 1 mg/mL BSA, 100 μM Cytidinediphosphocholine (CDP-Choline), and 100 μM AAG. The reaction was stopped by adding 500 μL of methanol. The assay procedure, extraction, purification, and determination of produced PAF concentration were performed as previously described [26]. The enzyme activity was expressed as pmol of synthesized PAF per minute per mg of total protein. All experiments were conducted in triplicates.

#### Lyso PAF-AT activity assay

Assay was performed as previously described [20]. Briefly, the reaction was carried out at 37°C for 30 minutes in a final volume of 200 μL containing 0.125 mg/mL protein, 50 mM Tris–HCl (pH 7.4), 0.25 mg/mL BSA, 20 μmol/L Lyso-PAF, and 200 μmol/L acetyl-CoA. The reaction was stopped by adding 500 μL of methanol and the extraction, purification, and determination of PAF was carried out as mentioned in the PAF-CPT assay [26]. The enzyme activity was expressed as pmol of synthesized PAF per min per mg of total protein. All experiments were conducted in triplicates.

#### Statistical analysis

The Wilcoxon non parametric statistic analysis for one sample was performed to compare the sample of TD patient with 12 healthy women. The statistic significance was established at 5% (p < 0.05) and the variables of control group are shown as median and percentiles (25% – 75%).

## Results

The patient’s lipid profile was typical for TD (Table 1).

**Table 1:** Lipid profile and the biochemical measurements of the TD patient

	**TD patient**	**Desirable range**
** *Total cholesterol * **	88 mg/dL	<200 mg/dL
** *Triglycerides * **	100 mg/dL	<150 mg/dL
** *HDL-cholesterol * **	2 mg/dL	>40 mg/dL
** *LDL-cholesterol * **	66 mg/dL	<130 mg/dL
** *Leukocytes number * **	4.8 K/μL	4.0-10 K/μL
** *Erythrocytes number * **	4.6 M/μL	3.9-4.9 M/μL
** *Platelets * **	117 K/μL	150-400 K/μL

HDL-cholesterol: High density lipoprotein-cholesterol.

LDL-cholesterol: Low density lipoprotein cholesterol.

**Figure 1:**
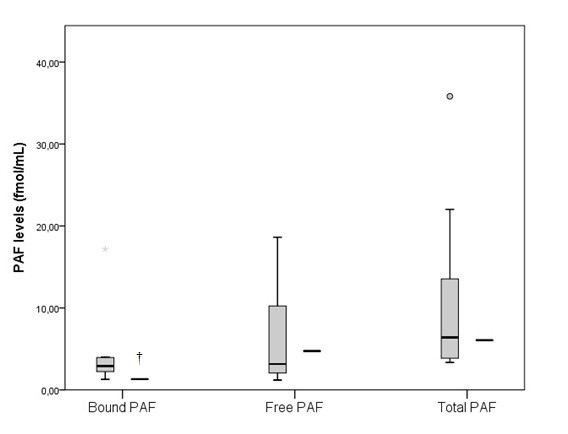
**Levels of Free-PAF, Bound-PAF and total PAF in blood of the control group and TD patient. **The box plots indicate the data from the control group while the single bar indicates the mean (and also median) value of TD patient. (†: statistically significant difference compared to control group, p < 0.05).

Patient’s “free” PAF was 4.74 fmol/mL and “bound” PAF was 1.32 fmol/mL giving a total PAF of 6.06 fmol/mL. In the control group “free” PAF was 3.16 fmol/mL (1.89-12.86) and “bound” PAF was 2.92 fmol/mL (2.27-3.99), so the total PAF was 6.40 fmol/mL (3.92-16.31) (Figure 1).

Many studies expressed the PAF levels in pg/mL and for this reason we converted our results from fmol/mL to pg/mL. After conversion with a theoretical molecular weight of PAF at 548 pg/mol, “free” PAF was 2.60 pg/mL and 1.73 pg/mL, “bound” PAF was 0.72 pg/mL and 1.60 pg/mL, and total PAF was 3.32 pg/mL and 3.50 pg/mL, in TD patient and control group, respectively.

**Figure 2:**
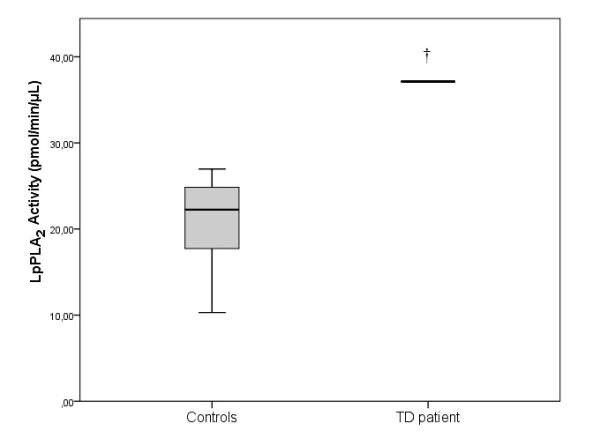
**The activity of Lipoprotein-phospholipase A**_**2**_**(Lp-PLA**_**2**_**) in plasma in TD patient and in control group. **The box plots indicate the data from the control group while the single bar indicates the mean (and also median) value of TD patient. (†: statistically significant difference compared to control group, p < 0.05).

**Figure 3:**
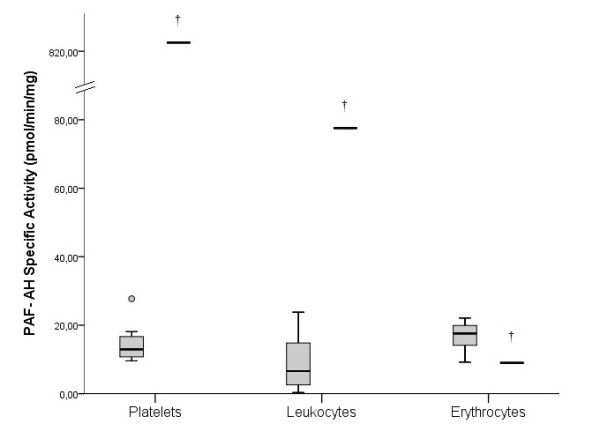
**The specific activity of PAF-acetylhydrolase (PAF-AH) in cells in TD patient and in control group. **The box plots indicate the data from the control group while the single bar indicates the mean (and also median) value of TD patient. (†: statistically significant difference compared to control group, p < 0.05).

**Figure 4:**
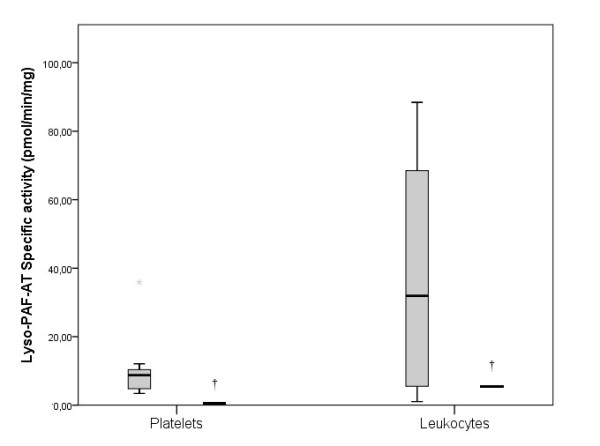
**The specific activity of Lyso-PAF-acelyltransferase (Lyso-PAF-AT) in cells, in TD patient and in control group. ** The box plots indicate the data from the control group while the single bar indicates the mean (and also median) value of TD patient. (†: statistically significant difference compared to control group, p < 0.05).

**Figure 5:**
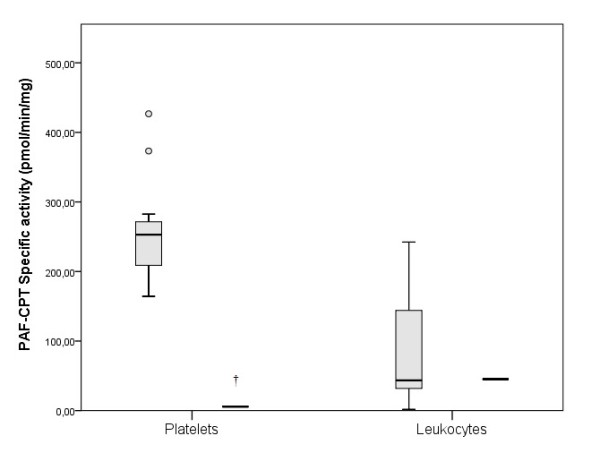
**The specific activity of PAF-cholinephosphotransferase (PAF-CPT) in cells, in TD patient and in control group. **The box plots indicate the data from the control group while the single bar indicates the mean (and also median) value of TD patient. (†: statistically significant difference compared to control group, p < 0.05).

The Lp-PLA_2_ activity was 37.12 pmol/min/μL in the TD patient, while in the control group was 22.25 pmol/min/μL (17.40-25.01) (p < 0.05) (Figure 2). The specific activity of PAF-AH in the TD patient’s leukocytes, erythrocytes and platelets was 77.56, 9.01 and 821.41 in pmol/min/mg of protein, respectively while in healthy women the corresponding activities were 6.60 (2.14-15.48), 17.26 (13.08-19.91), and 14.19 (11.74-17.92) (p < 0.05) (Figure 3). The activity of Lyso-PAF-AT in leukocytes and platelets was 5.52 and 0.37 pmol/min/mg of protein respectively, in the TD patient. In the control women the Lyso-PAF-AT specific activity in leukocytes was 20.31 (4.05-44.15) and in platelets was 9.18 (3.61-11.41) pmol/min/mg (p < 0.05) (Figure 4). PAF-CPT specific activity in leukocytes and platelets was 45.29 and 5.61 pmol/min/mg of protein, respectively, while in the control women’s leukocytes was 43.54 (33.88-121.14) and in platelets was 252.93 (212.36-282.48) pmol/min/mg (p < 0.05) (Figure 5).

**Figure 6:**
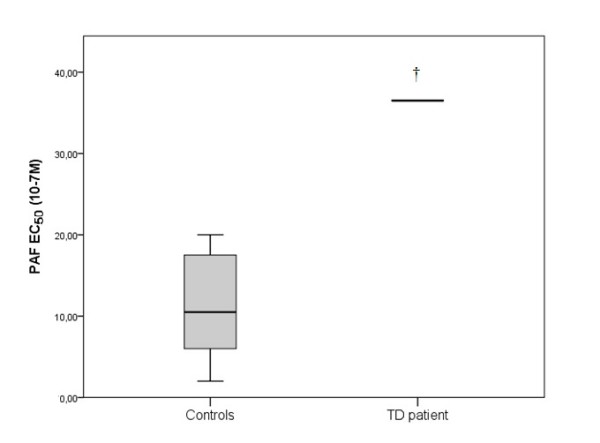
**The EC**_**50**_**of PAF in platelet rich plasma (PRP) in TD patient and in control group. **The box plots indicate the data from the control group while the single bar indicates the mean (and also median) value of TD patient. (†: statistically significant difference compared to control group, p < 0.05).

**Figure 7:**
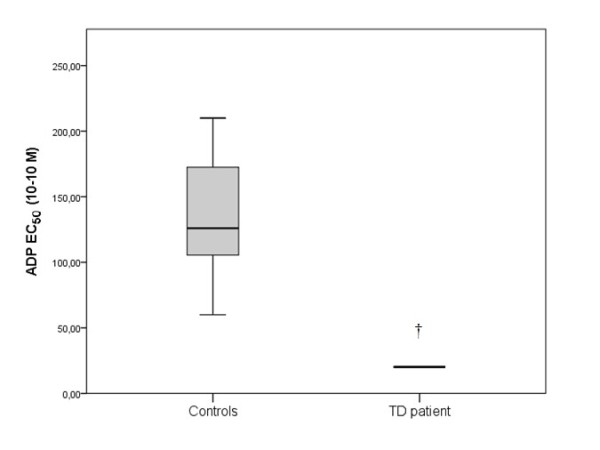
**The EC**_**50**_**of ADP in platelet rich plasma (PRP) in TD patient and in control group. **The box plots indicate the data from the control group while the single bar indicates the mean (and also median) value of TD patient. (†: statistically significant difference compared to control group, p < 0.05).

**Figure 8:**
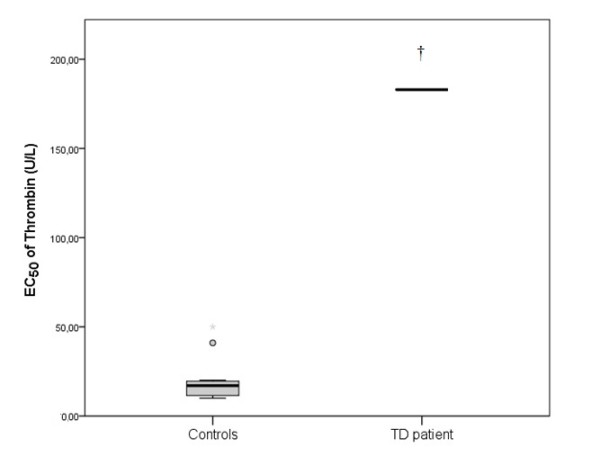
**The EC**_**50**_**of Thrombin in platelet rich plasma (PRP) in TD patient and in control group. **The box plots indicate the data from the control group while the single bar indicates the mean (and also median) value of TD patient. (†: statistically significant difference compared to control group, p < 0.05).

The patient’s EC_50_ value of the aggregation in PRP of PAF was 36.5·10^-7^ M, of ADP was 20.15·10^-10^ M and of Thrombin was 183·U/L (final concentration in the cuvette). In control group the EC_50_ of PAF was 9.5·10^-7^ (5-17) M, of ADP 126·10^-10^ (111-165) M and of thrombin 17 (12-20) U/L (Figures 6, 7 and 8).

## Discussion

### PAF levels

This is the first time that PAF levels were measured in blood sample of TD patient. Although, the bound PAF values of TD patient were lower in comparison with the control group, the total PAF values were in the normal range. Chen et al. [27] evaluated the PAF in nonsmoking patients with periodontits and/or CAD and found different values compared to our study. Also, different PAF values were found in few other studies evaluated patients with periodontal disease [28,29], asthma [30], anaphylaxis [31], heart failure [32] and liver cirrhosis [33]. In all of these studies the PAF values were higher compared to controls. The discrepancy found between our study and others investigators can be explained that we performed measurements with different method and the disease was different.

### Lp-PLA_2_, PAF-AH, Lyso-PAF-AT, PAF-CPT

This is the first study which evaluated the specific activities of PAF-AH, Lyso-PAF-AT, PAF-CPT and the activity of Lp-PLA_2_, in a TD patient. However, Pritchard et al. [34] and Stafforini et al. [35] have evaluated solely the Lp-PLA_2_ activity in patient with TD. Both studies found that the activity of Lp-PLA_2_ was higher in patients than controls. The values of the activities of Lp-PLA_2_, similar to PAF, differ among studies. This happens because the evaluated variables are measured in different samples (plasma or blood cells). Furthermore, in several pathological situations, such as acute coronary syndrome and unstable angina [9], polycystic ovary syndrome [36], heart failure [32], human immunodeficiency virus (HIV) infection and acquired immunodeficiency syndrome (AIDS) [37], CAD patients after positive exercise test [38] and hypertension [39], the Lp-PLA_2_ activity was increased compared with controls.

A number of studies evaluating the activity of Lp-PLA_2_ in various pathological situations found lower values compared with control group. Vadas et al. [31] measured the activity of Lp-PLA_2_ in patients with anaphylaxis and found lower specific activity than in control group and PAF values of 805 ± 595 pg/mL in patients and 127 ± 104 pg/mL in controls. Similar results were reported by Rufail et al. [40] in patients with generalized aggressive periodontitis and Lösche et al. [41] who studied patents with periodontitis before and after treatment. In addition, Serebruany et al. [42] measured plasma levels of PAF-AH and found that in patients with acute myocardial infarction were significantly lower comared with controls.

In our study the patient’s specific activities of PAF-AH in platelets and leukocytes and the activity of Lp-PLA_2_ in plasma, were increased compared to healthy women (p < 0.05). The patient’s specific activities of PAF-CPT in platelets and Lyso-PAF-AT in platelets and leukocytes, were decreased compared to healthy women (p < 0.05). The decreased specific activities of PAF-CPT and Lyso-PAF-AT also indicate the lack of a chronic or an acute inflammation.

### Platelets aggregation

It is the first time that EC_50_ of the aggregation in TD patient’s PRP of PAF, ADP and thrombin are measured. Stathopoulou et al. [43] found in 69 healthy individuals that the PAF EC_50_ values ranged 0.13-24.37 10^-7^ M. In addition, Goudevenos et al. [38] studied platelet response to the aggregatory effect of PAF in 44 patients with CAD who performed exercise tests. The PAF EC_50_ values in 21 patients with positive exercise test were found to be significantly decreased at rest compared with 21 subjects with negative exercise test.

In our study the patient’s EC_50_ of PAF was increased compared to healthy women (p < 0.05), which can be explained by the increased activity of Lp-PLA_2_. The patient’s EC_50_ values for ADP was lower and EC_50_ of Thrombin was higher, compared to healthy women (p < 0.05).

Tselepis et al. [44] studied platelet aggregation in the PRP of 32 patients with unstable angina before and after treatment with abciximab. The PAF EC_50_ values were significantly lower on the day of admission, whereas the maximal percentage of aggregation was significantly higher compared to controls. Similar behavior of the platelets was observed in the aggregatory effect of ADP. Also, Demopoulos et al. [45] found that ADP (5 μM) caused decreased or normal platelet aggregation in the homozygous beta-thalassaemic patients, approximately normal in the heterozygous subjects and increased in the splenectomized patients.

Harmon et al. [46] found that thrombin binding was elevated in TD patients but with lower responsiveness at lower thrombin concentrations and they suggested that platelets were less responsive to thrombin than in normal control subjects. We found higher value of EC_50_ of thrombin in TD patient compared with controls and we can assume that some co-enzymes could be in lower concentrations or less active in PRP of TD patient.

The reason why our TD patient has not developed foam cells and CAD is unknown and was extensively discussed by Kolovou et al. [17]. Furthermore, it may be partially explained by PAF metabolism. Since, PAF play an important role in atherosclerosis the development of CAD may depend on PAF levels in blood, as well as, biosynthesis and catabolism of PAF in cells.

In conclusion, the biosynthetic enzymes (PAF-CPT and Lyso-PAF-AT) of TD patient have lower specific activity than of the control group, which can be explained by the decreased levels of PAF. The catabolic enzyme PAF-AH specific activity in leukocytes, in platelets and Lp-PLA_2_ activity in plasma is higher in TD patient than in controls. Opposite, the PAF-AH in erythrocytes was lower than in control group.

Thus, this may partially explain why bound PAF levels are lower in TD patient compared with normal subjects. The measurements of total PAF levels which were close to the normal range, may be the reason for not developing CAD in TD patients.

## Limitations

It is hard to establish whether the features observed with this TD patient are similar in other TD patients, since TD is a rare genetic disease with approximately 50 indentified cases all over the world. In addition, this is the first time that PAF metabolism, as well as, PAF levels and aggregability, were measured in a TD patient.

## Competing interests

The authors declare that they have no competing interests.

## Authors’ contributions

VK participated in the experiments procedures and drafting of the manuscript. VP, GS, SV and MX participated in the experiments procedures and review the manuscript. GK evaluated the patient and review the manuscript. CD participated in the study design, its coordination and review the manuscript. All authors read and approved the final manuscript.
